# Explaining the Association Between Number of Teeth and Frailty in Older Chinese Adults: The Chain Mediating Effect of Nutritional Status and Cognitive Function

**DOI:** 10.1111/ijn.70001

**Published:** 2025-02-19

**Authors:** Yanan Wang, Lifang Fan, Hui Zhou, Meimei Zhang, Tianyan Wang, Ying Sheng, Yuelan Zhu

**Affiliations:** ^1^ Nursing Department The First People's Hospital of Kunshan Suzhou China; ^2^ Scientific Research Department Shanghai Fifth People's Hospital, Fudan University Shanghai China

**Keywords:** cognitive function, frailty, number of teeth, nutritional status, older adults

## Abstract

**Aims:**

The objective of this research is to investigate the association between the number of teeth and frailty in the geriatric population.

**Design:**

This cross‐sectional study was conducted in Shanghai Fifth People's Hospital, Fudan University, from May 2021 to September 2021.

**Methods:**

A cohort of 538 community‐dwelling older adults aged 60 years or above was included in the analysis. The Tilburg Frailty Indicator, Mini‐Mental State Examination and Mini Nutritional Assessment Short‐Form were employed to assess frailty, cognitive function and nutritional status, respectively. The statistics software SPSS v21.0 and its process plug‐in were employed for statistical analysis, and the significance of intermediary effects was tested using the bootstrap sampling test method and the process plug‐in.

**Results:**

The number of teeth influenced frailty through three mediating pathways: (a) nutritional status (effect = −0.038); (b) cognitive function (effect = −0.021); (c) nutritional status and cognitive function combined (effect = −0.038). The total mediating effect accounted for 50.26% of the overall effect.

**Conclusion:**

These findings highlight the need to raise awareness of oral health care among older adults and implement comprehensive interventions to promote active aging and improve their overall well‐being.

**Abbreviations**
BMI: body mass index
CF: cognitive function
DE: direct effect
IE: indirect effect
MMSE: Mini‐Mental State Examination
MNA‐SF: Mini Nutritional Assessment Short‐Form
NoT: number of teeth
NS: nutritional status
TE: total effect
TFI: Tilburg Frailty Index

**Summary**

**What is already known about this topic?**

- Recent research suggests that frailty may be associated with a higher risk of tooth loss, independent of age, sex and socioeconomic status.
- Furthermore, emerging evidence suggests a potential bidirectional relationship between oral health, nutritional status and cognitive function.
- No studies have been conducted to examine and comprehend the relationships between frailty, number of teeth, nutritional status and cognitive function in mainland China.

**What this paper adds?**

- Nutritional status plays a mediating role in the relationship between the number of teeth and frailty.
- Cognitive function serves as a mediator between the number of teeth and frailty.
- A chain mediating mechanism was identified, where nutritional status had a significantly greater mediating effect compared with both cognitive function and the chain mediation pathways.

**The implications of this paper:**

- Based on the findings from this study, nurses can assume a pivotal role in clinical practice. They can provide vital contributions by delivering oral health education, assessing nutritional status and cognitive function, and offering support and interventions to mitigate the impact of tooth loss on frailty. Consequently, such nurse‐led interventions can significantly enhance the overall well‐being and quality of life for individuals affected by these factors.
- The study's findings contribute to a deeper understanding of the complex interplay between oral health, nutritional status, cognitive function and frailty. This underscores the significance of oral health in overall health management and may prompt increased research and attention in practice.

## 1 Introduction

As the global population continues to age, especially in China, where the proportion of individuals aged 60 or older reached 17.9% by the end of 2018 (Zhao et al. 2021), it is expected to increase to approximately 35.1% by 2050 (Ma et al. 2022). With the growing population of older adults, there is a noticeable shift in research and clinical practice towards frailty (Ma et al. 2022; Nagai et al. 2020). Frailty is a multidimensional geriatric syndrome characterized by age‐related declines in the regulatory functions of the body. It involves cumulative damage across multiple systems, including diminished muscle strength, age‐related hormonal imbalances, inflammation, and disruptions in nutrition and metabolism. These impairments lead to a decrease in physiological reserve capacity, affecting physical activity, energy metabolism and cognitive level among older adults (Komatsu et al. 2021; Rezaei‐Shahsavarloo et al. 2020; Zhao et al. 2021). Frailty not only increases the risk of falls, disability, impairment, delirium, hospitalization and mortality in older adults but also poses a significant threat to their overall health status and life expectancy. Early screening, intervention programmes and reducing risk factors are key strategies to prevent or delay frailty in older adults (Huang et al. 2023).

According to the World Dental Federation, oral health encompasses the ability to engage in activities such as smiling, speaking and laughing without experiencing any disorders or facing social or psychological challenges (Zhang, Wu, and Chen 2022). The most prevalent and consequential oral diseases include untreated dental caries, periodontal diseases, tooth loss and oral cancer (Winkelmann, Gomez, and van Ginneken 2022). As people experience longer lifespans and maintain more of their natural teeth in older age, oral health has become increasingly important for enhancing the overall quality of life (Toyama et al. 2021). In fact, a number of cross‐sectional and cohort studies have investigated how oral health is related to frailty among the geriatric population, and the findings have shown that tooth loss can contribute to an increased risk of frailty (Dibello et al. 2021; Iwasaki et al. 2018; Kuo et al. 2021; Zhang, Jiao, et al. 2022). A recent study conducted in 2021 found that physical frailty was negatively associated with an individual's number of teeth (NoT) (Kuo et al. 2021). In addition, a 3‐year cohort study also demonstrated that each additional tooth in older adults was associated with a 5% reduction in the prevalence of frailty (Castrejon‐Pérez et al. 2017). Contrary to previous findings, a study conducted in Finland on older adults demonstrated no significant difference in NoT between the frail and non‐frail groups (Saarela et al. 2021). Therefore, the relationship between NoT and frailty among older adults remains a topic of ongoing research.

Previous studies have suggested a correlation between nutritional status (NS) and NoT (Shin 2020; Su et al. 2020). There is no doubt that tooth loss can affect masticatory function and lead to dietary restrictions, including a decreased intake of fruits and vegetables, which are essential for maintaining good NS (Xia et al. 2022). Numerous studies have confirmed the relationship between NS and frailty. Adequate nutrition plays a crucial role in maintaining overall health and preventing the onset of frailty among older adults (Huang et al. 2020; Lochlainn et al. 2021). Xia et al. (2022) highlight the potential impact of NoT on the intake of nutritious food and its association with malnutrition, ultimately leading to muscle loss. Moreover, they suggest that NS plays a partial mediating role in the association between NoT and sarcopenia (Xia et al. 2022). Studies have consistently shown that NoT can have an impact on NS, and NS, in turn, has been found to be a predictor of frailty. Therefore, we can infer that the relationship between NoT and frailty is mediated, at least in part, by NS.

In recent years, mounting evidence suggests that older adults exhibit lower cognitive performance in the presence of certain oral health conditions, especially periodontal disease and tooth loss (Nangle et al. 2019; Yang et al. 2022; Qi et al. 2021). Kang et al. emphasized that diminished cognitive function (CF) in the early stages was associated with poorer oral health and an elevated risk of tooth loss in later life (Kang et al. 2019). A cross‐sectional analysis of data from the Bushehr Elderly Health Program revealed a potential association between cognitive impairment and frailty, with a stronger link observed between cognitive impairment and low muscle strength and function (Sharifi et al. 2021). Additionally, a prospective cohort study based on the Chinese Longitudinal Healthy Longevity Study 2011 wave database demonstrated that cognitive impairment emerged as a significant predictor of frailty in older adults (Zhao et al. 2022). This evidence supports the hypothesis that CF mediates the association between NoT and frailty.

Indeed, NS and CF are closely intertwined, and their relationship could be bidirectional. Hsu et al. performed a cohort study in older men and showed that the risk of malnutrition was significantly associated with 3‐year cognitive decline (Hsu et al. 2019). A recent study by Liu et al. showed that the negative effects of cognitive decline on sarcopenia were mediated by NS (Liu et al. 2021). A randomized study demonstrated that the increase in dietary intake, especially in terms of energy and protein density, had an effect on older adults' CF (Blondal et al. 2022; Donders et al. 2020). Hence, we assume that NS and CF can act as chain mediators in the association between NoT and frailty.

Nurses, as the largest group within the healthcare workforce, play a crucial role in implementing health promotion and preventive strategies for older adults (Flyum et al. 2022). Studies have underscored the essential role of nurses in identifying various factors contributing to frailty, such as disability, poor nutrition and dementia, as well as their ability to reverse or mitigate frailty and its adverse effects through the implementation of multifaceted interventions (Gobbens et al. 2022; Kasa, Traynor, and Drury 2024). For instance, a systematic review by Kasa et al. (2023) demonstrated that nurse‐led interventions, including cognitive behavioural therapy, exercise programmes, nutritional intervention and education, effectively improved physical capabilities and mental health outcomes in frail older adults. Furthermore, a systematic review suggests that multifaceted interventions designed by nurses should comprehensively address the multiple underlying factors associated with frailty in older individuals to optimize their health and well‐being (Zheng et al. 2024).

**FIGURE 1:**
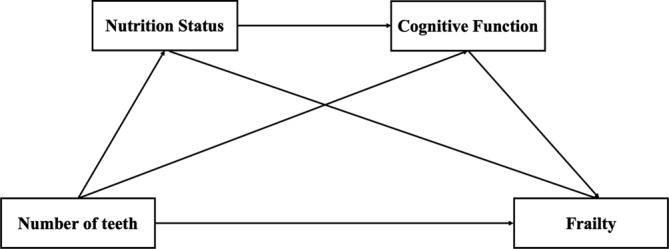
Conceptual framework.

To sum up, the impact of tooth loss on frailty is a subject of ongoing debate, and the specific mechanisms underlying this relationship remain unclear. To our knowledge, no study has integrated NoT, NS, CF and frailty into a comprehensive framework. Moreover, there is a lack of relevant research in the Chinese context. To address these knowledge gaps, we examined the pathway through which NoT influences frailty and constructed a chain of mediating models, as depicted in Figure 1. The findings aided nurses to develop targeted and multifaceted interventions.

## 2 Methods

### 2.1 Aim(s)

This study aims to investigate the relationship between NoT and frailty among Chinese older adults and examine if NS and CF mediated the association between NoT and frailty.

### 2.2 Design

A cross‐sectional survey was performed from May 2021 to September 2021 through a convenience sampling method in Shanghai Fifth People's Hospital, Fudan University.

### 2.3 Participants

According to the formula for sample size, *N* = [*Z*_*α/2*_^2^ *p q*]/*δ*, where *p* is 60% (Song et al. 2020), *α* = 0.05 and *δ* = 0.1 × *p*, the sample size required for this research is 267. Considering a design effect of 1.5, the final sample size was adjusted to 401 (Zegeye, Temachu, and Mekonnen 2023), resulting in a minimum final sample size of 461 participants, accounting for an anticipated 15% attrition rate and potential missing data. The study included participants who met the following inclusion criteria: (i) adults aged 60 years and above and (ii) willingness and ability to participate in the investigation. The participants were excluded if they met any of the following exclusion criteria: (i) brain trauma or space‐occupying lesions and other neurological disorders that may affect cognition, such as dementia, Parkinson's disease or multiple sclerosis; (ii) diagnosis of psychosis; (iii) severe physical illness (such as decompensated heart, liver or kidney function); and (iv) significant hearing or visual impairments.

Five hundred and fifty questionnaires were distributed, out of which 538 valid questionnaires were collected, resulting in an effective recovery rate of 97.82%.

### 2.4 Data Collection

A standardized questionnaire was conducted, and nurses involved in the study received uniform training. After obtaining patient consent, the survey questionnaire was distributed. For those unable to complete the survey personally, nurses used consistent language to read and explain the items, assisting them in filling out the questionnaire. General information such as gender, age and medical history was obtained by reviewing electronic medical records.

### 2.5 Measure

The assessment of the number of natural teeth involved two steps. First, participants provided self‐reported information by answering the question, ‘How many natural teeth do you still have?’ (excluding false teeth). Secondly, a dental examination was conducted by two trained nurses under artificial light. In the event of any discrepancies in the count results, an experienced dentist was invited to conduct a re‐examination, ensuring that a consensus was ultimately reached. Tooth loss counts were recorded as the number of missing teeth (Hao et al. 2023). The range of remaining teeth varied from 0 to 32 (Donders et al. 2020).

Frailty was evaluated using the Tilburg Frailty Index (TFI), which contains 15 items addressing frailty from the physical (eight items), psychological (four items) and social (three items) aspects (Song et al. 2020). The TFI scored between 0 and 15, with ≥ 5 indicating frailty. A Cronbach's α of 0.71 was used to validate the TFI among the Chinese older population (Qin et al. 2021).

CF was examined using the Mini‐Mental State Examination (MMSE). The MMSE is a 30‐point test for evaluating orientation, registration, attention and calculation, and memory and language (Wu et al. 2020). Each correct answer on the MMSE is assigned one point, resulting in a total score of 30 points. Among participants, illiteracy of ≤ 21, primary school of ≤ 24 and middle school and above of ≤ 27 were diagnosed as MCI (Cui et al. 2021). The Cronbach's alpha coefficient for the Chinese version of the MMSE was 0.902, indicating good internal consistency (Li et al. 2022).

NS was evaluated using the Mini Nutritional Assessment Short‐Form (MNA‐SF). The MNA‐SF is a six‐item scale, and responses yield a score from 0 to 14 points. Based on the total weighted MNA‐SF scores, 12–14 indicate normal NS, 8–11 indicate malnutrition risk and ≤ 7 indicate malnutrition (Krzymińska‐Siemaszko et al. 2020). The MNA‐SF has been validated in Chinese older adults, and the sensitivity and specificity were 89.6% and 88%, respectively (Lei et al. 2009).

### 2.6 Covariates

Previous studies have identified various factors associated with frailty. In our analysis, we included several variables related to sociodemographic factors, lifestyle behaviours and health status. Regarding sociodemographic variables, we considered age (in years), sex (male = 1, female = 2), marital status (married = 1, single, widowed or divorced = 2), education level (non‐formal education = 1, elementary education = 2, middle school education = 3, high school education = 4, university education or above = 5), living arrangement (live alone = 1, household/institution = 2) and primary occupation before retirement (professional work = 1, non‐professional work = 2). Lifestyle behaviours were assessed through variables such as recent drinking (never = 0, former = 1, current = 2), smoking (never = 0, former = 1, current = 2) and engagement in physical exercises (never = 0, 1–2 times/week = 1, ≥ 3 times/week = 2). Body mass index (BMI) was calculated by dividing weight (kg) by height (m^2^). Self‐reported chronic diseases encompassed diabetes, hypertension, cerebrovascular disease, stroke, heart disease, chronic obstructive pulmonary disease, chronic gastroduodenal ulcer and cancers. The analysis incorporated the total number of chronic diseases reported by the participants.

### 2.7 Ethical Considerations

Ethical approval for this study was obtained from the Medical Ethics Committee of Shanghai Fifth People's Hospital, Fudan University (No.: 2020.168). All participants provided informed consent prior to their involvement in the study.

### 2.8 Data Analysis

The data were analysed with SPSS v21.0 software (SPSS Inc., United States). Continuous data were described by means (standard deviation [SD]), whereas categorical data were described by numbers and percentages. Spearman's coefficients were employed to assess the correlations among the variables. To examine the chain mediation model, bias‐corrected percentile bootstrap analysis was selected as the appropriate statistical method (Hou et al. 2020). To analyse the chain mediation mechanism, the PROCESS 4.0 macro for SPSS, Model 6, was utilized (Wang, Xie, and Xu 2022). The number of bootstrap samples was fixed at 5000. In all the analyses, the covariates included age, smoking, drinking, physical exercise and self‐reported chronic diseases. *p* < 0.05 was deemed statistically significant. When the 95% CI did not overlap with zero, a significant mediating effect was observed (Xiong and Xue 2022).

## 3 Results

### 3.1 Characteristics of the Participants

Our study enrolled 538 older adults, with a mean age of 71.05 ± 7.90 years. Among them, 51.1% were men, and 82.3% were married. In terms of NoT, the mean score was 16.52 (range: 0–32). In total, approximately 42.2% of older adults were found to be frail. Table 1 provides additional information regarding the variables included in the study and their descriptive statistics.

**TABLE 1:** Characteristics of all participants.

Variable	Mean ± standard deviation or *n* (%)	Variable	Mean ± standard deviation or *n* (%)
Age (years)	71.05 ± 7.90	Drinking	
Sex		Never	284 (52.8)
Male	275 (51.1)	Former	148 (27.5)
Female	263 (48.9)	Current	106 (19.7)
Marital status		Physical exercise (time/week)	
Married	443 (82.3)	0	155 (28.8)
Single, widowed or divorced	95 (17.7)	1–2	184 (34.2)
Education level		≥ 3	199 (37.0)
Non‐formal education	40 (7.4)	BMI (kg/m^2^)	23.21 ± 3.46
Elementary education	142 (26.4)	Self‐reported chronic disease	2.70 ± 1.11
Middle school education	195 (36.2)	NoT	16.52 ± 9.61
High school education	125 (23.2)	Frailty	
University education or above	36 (6.7)	Non‐frail	311 (57.8)
Living arrangement		Frail	227 (42.2)
Live alone	66 (12.3)	NS	
Household/institution	472 (87.7)	Normal NS (12–14)	356 (66.2)
Primary occupation before retirement		Malnutrition risk (8–11)	131 (24.3)
Professional work	350 (65.1)	Malnutrition (≤ 7)	51 (9.5)
Non‐professional work	188 (34.9)	CF	
Smoking		Normal	295 (54.8)
Never	341 (63.4)	Abnormal	243 (45.2)
Former	98 (18.2)		
Current	99 (18.4)		

Abbreviations: BMI, body mass index; CF, cognitive function; NoT, number of teeth; NS, nutritional status.

### 3.2 Correlation Analysis of Variables

Table 2 presents the correlations between frailty and variables. There were significant negative correlations among the degree of frailty and NoT (*r* = −0.562, *p* < 0.001), NS (*r =* −0.567, *p* < 0.001) and CF (*r* = −0.588, *p* < 0.001). Moreover, NoT was positively associated with NS (*r =* 0.630, *p* < 0.001) and CF (*r =* 0.644, *p* < 0.001). Similarly, NS was positively associated with CF (*r =* 0.806, *p* < 0.001).

**TABLE 2:** Correlations among key variables (*n* = 538).

Variable	Mean	SD	1.	2.	3.	4.
1. Frailty	4.19	3.43	1	—	—	—
2. NoT	16.52	9.61	−0.562**	1	—	—
3. NS	11.7	2.46	−0.567**	0.630**	1	—
4. CF	24.74	5.268	−0.588**	0.644**	0.806**	1

Abbreviations: CF, cognitive function; NoT, number of teeth; NS, nutritional status; SD, standard deviation.

** *p* < 0.001.

### 3.3 Chain Mediating Effects Analysis

**FIGURE 2:**
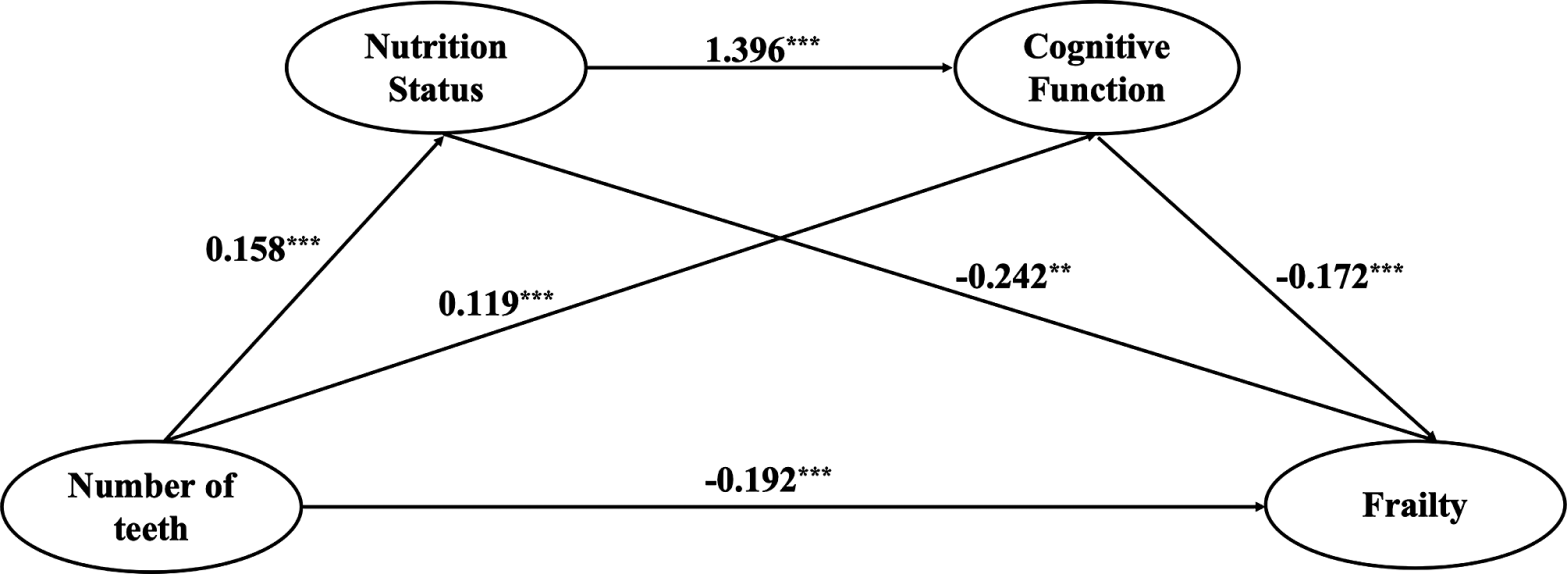
The chain mediating effect of nutrition status and cognitive function. **p < 0.01 and ***p < 0.001.

Next, we investigated the potential mediation of NS and CF in the association between NoT and frailty using the PROCESS macro for SPSS. Before conducting the regression analysis, a multicollinearity test was executed on the variables and confirmed that the variance inflation factor (VIF) values were all below 5, which aligns with the specified criteria. The outcomes of this analysis are summarized in Table 3 and Figure 2. According to the results in Table 3: In Equation 1, NoT positively influenced NS (*b* = 0.158, *p* < 0.001). In Equation 2, CF was positively affected by NoT (*b* = 0.119, *p* < 0.001) and NS (*b* = 1.396, *p* < 0.001). In Equation 3, NoT (*b* = −0.096, *p* < 0.001), NS (*b* = −0.242, *p* < 0.01) and CF (*b* = −0.172, *p* < 0.001) negatively influenced frailty. In Equation 4, NoT (*b* = −0.192, *p* < 0.001) negatively affected frailty.

**TABLE 3:** Regression data of the chain mediation effect mechanism (*n* = 538).

Outcome variable	Predictive variable	*R* ^2^	*F*	*b*	SEs	*t*	LLCI	ULCI
Equation 1								
NS	NoT	0.527	118.381***	0.158***	0.008	18.716	0.141	0.174
Equation 2								
CF	NoT	0.758	277.563***	0.119***	0.017	7.177	0.087	0.152
	NS			1.396***	0.067	20.979	1.265	1.526
Equation 3								
Frailty	NoT	0.558	94.470***	−0.096***	0.015	−6.237	−0.126	−0.066
	NS			−0.242**	0.079	−3.057	−0.398	−0.087
	CF			−0.172***	0.038	−4.515	−0.248	−0.096
Equation 4								
Frailty	NoT	0.484	99.784***	−0.192***	0.012	−15.688	−0.217	−0.168

*Note:* The estimated coefficients are non‐standardized. Adjusted variables include age, smoking, drinking, physical exercise, and self‐reported chronic diseases.

Abbreviations: CF, cognitive function; LLCI, lower limit of the 95% CI; NoT, number of teeth; NS, nutritional status; ULCI, upper limit of the 95% CI.

** *p* < 0.01.

*** *p* < 0.001.

As shown in Figure 2, NS and CF played a mediation role in the association between NoT and frailty. Notably, four paths were identified to be responsible for the association between NoT and frailty: (a) NoT → frailty; (b) NoT → NS → frailty; (c) NoT → CF → frailty; and (d) NoT → NS → CF → frailty.

As a result, the presence of a chain mediation effect mechanism was confirmed by the significant path (d).

### 3.4 Total Effect (TE), Direct Effect (DE) and Indirect Effect (IE) of the Chain Mediation Mechanism

As shown in Table 4, the TE, DE and IE of the chain mediation mechanism were calculated. It was found that the total IE (−0.097) accounted for 50.26% of the TE (−0.193) and 100% of the DE (−0.096) in the association between NoT and frailty. These findings indicate that 50.26% of the adverse impact of NoT on frailty is mediated through three distinct mechanisms. Notably, these mechanisms include (a) the mediation effect of NS, (b) the mediation effect of CF and (c) the combined mediation effects of NS and CF. According to Table 4, the mediation effects (a), (b) and (c) accounted for 19.69%, 10.88% and 19.69% of the TE, respectively, and 39.58%, 21.88% and 39.58% of the DE, respectively. All tests conducted for the TE, DE and IE, as well as the mediation effects of (a), (b) and (c), were statistically significant at 95% CIs that did not overlap with zero.

**TABLE 4:** Comparison of chain mediation effects (*n* = 538).

	Effect	Boot *SE*	Boot LLCI	Boot ULCI	IE‐to‐TE ratio	IE‐to‐DE ratio
TE	−0.193	0.012	−0.217	−0.168	—	—
DE	−0.096	0.015	−0.126	−0.066	—	—
IE	−0.097	0.012	−0.119	−0.074	50.26%	100%
lnd1	−0.038	0.014	−0.065	−0.011	19.69%	39.58%
lnd2	−0.021	0.006	−0.034	−0.010	10.88%	21.88%
lnd3	−0.038	0.010	−0.058	−0.020	19.69%	39.58%

*Note:* Ind1 is the mediation effect model (MEM) of NoT → NS → frailty, Ind2 is the MEM of NoT → CF → frailty, and Ind3 is the MEM of NoT → NS → CF → frailty. The estimated SE through bias corrected percentile bootstrap approach, lower 95% CI, and upper 95% CI stand for Boot SE, Boot LLCI, and Boot ULCL, respectively. Both Boot LLCI and Boot ULCL exhibit non‐overlap with zero.

## 4 Discussion

To our knowledge, no research has been conducted to investigate the mechanisms underlying the association between NoT and frailty in Chinese older adults. Specifically, this study proposes a significant and negative association between NoT and frailty, which aligns with previous research findings. Moreover, in line with our hypotheses, the results indicate that NoT indirectly affects frailty through factors such as NS and CF, with NS and CF acting as mediating variables in this relationship. The overall mediation effect accounts for 50.26%, highlighting the crucial impact of mediators on the association between NoT and frailty. The mechanism through which NoT contributes to frailty has been previously elucidated and involves three potential pathways: nutritional, psychological and inflammatory factors (Zhang, Jiao, et al. 2022). Tooth loss in older adults, particularly edentulism without false teeth, can diminish chewing ability, influencing their dietary intake and potentially resulting in malnutrition and frailty (Xu et al. 2023). In addition, severe tooth loss may lead to facial deformities that affect language, social image and self‐esteem, with potential psychosocial effects. These changes can limit social interactions, leading to cognitive or mental disorders and an increased risk of frailty (Xu et al. 2023; Zhang, Wu, and Chen 2022). Studies have revealed that periodontitis and oral inflammation are major contributors to tooth loss in later life, thereby increasing the risk of cerebrovascular disease and potentially causing functional disability in older adults (Hao et al. 2023; Xu et al. 2023).

In this study, NS was identified as a significant mediator in the relationship between NoT and frailty, accounting for 39.58% of the total effect. This mediation effect suggests that NS plays a crucial role in the development of frailty among older adults with tooth loss. Specifically, tooth loss impairs chewing ability, leading to reduced dietary intake and poor NS (Hakeem, Bernabe, and Sabbah 2019; Kang and Jung 2022). As given that malnutrition is a known risk factor for frailty, NS may serve as a critical pathway through which NoT influences frailty (Lochlainn and Robinson 2021). Furthermore, the mediating effect of NS underscores the importance of addressing nutrition‐related issues in older adults with tooth loss (Hakeem, Bernabe, and Sabbah 2021; Kim, Lee, and Lee 2022). Improving dietary intake, ensuring proper nutrient absorption and promoting interventions to manage malnutrition could therefore be effective strategies to mitigate the risk of frailty in this population.

This study reveals that CF serves as a mediator in the relationship between NoT and frailty, with a mediation effect of 21.88%. This suggests that individuals with fewer teeth are at a higher risk of developing frailty, especially when CF is impaired. These findings are consistent with previous research that has demonstrated a positive association between NoT and CF, with cognitive impairment acting as a significant risk factor for frailty (Yun et al. 2020; Nagatani et al. 2023). Furthermore, studies focusing on oral health in frail older adults have identified oral problems as key risk factors for cognitive decline within this population and have underscored the substantial oral health challenges faced by frail older adults (Dibello et al. 2021; Saarela et al. 2021; Turnbull et al. 2022). Collectively, these findings deepen our understanding of the complex interplay between oral health, CF and frailty, highlighting the critical importance of preserving CF in frailty management for older adults.

In addition, this study highlights the interconnected mediation effect of NS and CF in the relationship between NoT and frailty. Specifically, it reveals that older adults who experience tooth loss are more likely to develop frailty due to poor NS and a lower level of CF. Notably, malnutrition and cognitive decline can influence each other in a reciprocal manner (Yu et al. 2021). Malnutrition can impact CF through various mechanisms (Gutierrez et al. 2021), and conversely, CF can also affect NS (Xiong and Xue 2022). Research has shown that weight loss and malnutrition are prominent symptoms in individuals with dementia and can be observed during the early stages of Alzheimer's disease (Yu et al. 2021; Smith and Greenwood 2008). Numerous studies have proposed that adopting a healthy dietary management strategy, encompassing factors such as food choices, dietary patterns and dietary supplements, may prove effective in preventing cognitive impairment and reducing the prevalence of frailty in older adults (Gutierrez et al. 2021; Buckinx and Aubertin‐Leheudre 2021; Huang et al. 2020). These findings underscore the importance of considering both NS and CF in interventions aimed at preventing frailty and promoting the overall well‐being of older individuals.

This study emphasizes the critical role of oral health in the prevention and management of frailty. In 2020, the Registered Nurses' Association of Ontario (RNAO) published the ‘Oral Health: Supporting Adults Who Need Help (2nd edition)’ clinical practice guideline, which offers comprehensive recommendations for oral health assessment, the development of oral care plans, health education and the promotion of interdisciplinary collaborative care (Registered Nurses’ Association of Ontario 2020). Additionally, nutritional support and cognitive training have been identified as critical components in mitigating the effects of tooth loss and frailty. Our study suggests that interventions incorporating nutritional support, cognitive training and oral health care can significantly mitigate the negative consequences of frailty.

A 12‐month, multicenter, randomized controlled trial indicates that educational programmes focusing on physical activity, nutrition, cognitive training and psychosocial well‐being could be an easy‐to‐implement strategy within healthcare systems, especially for nurses, successfully maintaining functional independence of frail/pre‐frail older adults (González‐Mariscal et al. 2024). Nurses, being on the frontline of patient care and integral members of interdisciplinary healthcare teams, play a pivotal role in implementing these guidelines and promoting healthier aging. Further research is essential to continue refining these strategies and advancing nursing practice in this critical area, ensuring that the frail older adults receive the comprehensive care they require to maintain their quality of life.

Nonetheless, this study has several limitations that should be acknowledged.

Firstly, it should be noted that this study adopts a cross‐sectional design, which limits its ability to establish causal relationships between the variables under investigation. Future research should employ longitudinal designs to investigate causal associations. Secondly, the variables such as ‘nutritional status’, ‘cognitive function’ and ‘frailty’ relied on self‐reported data provided by the participants, which may introduce information bias and affect the accuracy of the findings. Thirdly, the potential influence of confounding factors on the study outcomes could not be completely ruled out due to limitations in the available data. It is necessary to consider other factors that may affect the relationship between NoT, NS, CF and frailty in future studies. Finally, the sample used in this study was derived solely from a single province in China. Given the variations in lifestyle, economy and social culture across different regions, our results should be interpreted with caution. Further research should include diverse samples to enhance the external validity and generalizability of the research results.

## 5 Conclusions

The present study examined the mediation roles of NS and CF between NoT and frailty in the geriatric population. The findings indicate that NoT has a direct negative association with frailty, as well as an indirect association mediated by NS and CF. Notably, this study identified the following mediating pathway: NoT → NS → CF → frailty. NS and CF play crucial roles in the association between NoT and frailty. These findings underscore the need for integrated interventions that address not only nutritional support and cognitive training but also oral health care to mitigate the effects of frailty. By doing so, we can potentially contribute to a more effective frailty management strategy and enhance the well‐being of older adults.

## Author Contributions

Y.N.W. was responsible for study design. H.Z. and L.F.F. performed a literature search. Y.S. conducted data collection. M.M.Z. and T.Y.W. executed data analysis. Y.N.W. wrote the manuscript. Y.L.Z. provided the final version for submission. The authors have read and approved the final manuscript.

## Ethics Statement

Ethical approval for this study was obtained from the Medical Ethics Committee of Shanghai Fifth People's Hospital, Fudan University (No.: 2020.168). All participants provided informed consent prior to their involvement in the study.

## Consent

The authors have nothing to report.

## Conflicts of Interest

The authors declare no conflicts of interest.

## Data Availability

Data sharing is not applicable to this article as no new data were created or analyzed in this study.

## References

[ijn70001-bib-0001] Blondal, B. S. , O. G. Geirsdottir , T. I. Halldorsson , A. M. Beck , P. V. Jonsson , and A. Ramel . 2022. “HOMEFOOD Randomised Trial—Six‐Month Nutrition Therapy Improves Quality of Life, Self‐Rated Health, Cognitive Function, and Depression in Older Adults After Hospital Discharge.” Clinical Nutrition ESPEN 48: 74–81. 10.1016/j.clnesp.2022.01.010. 35331537

[ijn70001-bib-0002] Buckinx, F. , and M. Aubertin‐Leheudre . 2021. “Nutrition to Prevent of Treat Cognitive Impairment in Older Adults: A GRADE Recommendation.” Journal of Prevention of Alzheimer's Disease 8, no. 1: 110–116. 10.14283/jpad.2020.40. 33336232

[ijn70001-bib-0003] Castrejon‐Pérez, R. C. , A. Jiménez‐Corona , E. Bernabé , et al. 2017. “Oral Disease and 3‐Year Incidence of Frailty in Mexican Older Adults.” Journals of Gerontology. Series A, Biological Sciences and Medical Sciences 72, no. 7: 951–957. 10.1093/gerona/glw201. 28329793

[ijn70001-bib-0004] Cui, G. H. , S. J. Li , Q. Y. Kong , et al. 2021. “Association of Sleep Quality, Depressive Symptoms and Their Interaction With Cognitive Frailty in Elderly People (Chinese).” Chinese General Practice 24, no. 9: 1076–1081. 10.12114/j.issn.1007-9572.2021.00.118.

[ijn70001-bib-0005] Dibello, V. , R. Zupo , R. Sardone , et al. 2021. “Oral Frailty and Its Determinants in Older Age: A Systematic Review.” Lancet Healthy Longevity 2, no. 8: e507–e520. 10.1016/S2666-7568(21)00143-4. 36098000

[ijn70001-bib-0006] Donders, H. C. M. , L. M. IJzerman , M. Soffner , A. W. J. Hof , B. G. Loos , and J. D. Lange . 2020. “Elevated Coronary Artery Calcium Scores Are Associated With Tooth Loss.” PLoS ONE 15, no. 12: e0243232–e0243243. 10.1371/journal.pone.0243232. 33326424 PMC7743922

[ijn70001-bib-0007] Flyum, I. R. , E. R. Gjevjon , A. Josse‐Eklund , E. Lærum‐Onsager , and G. Borglin . 2022. “Nursing, Frailty, Functional Decline and Models of Care in Relation to Older People Receiving Long‐Term Care: A Scoping Review Protocol.” BMJ Open 12, no. 8: e061303–e061310. 10.1136/bmjopen-2022-061303. PMC9403107 35998956

[ijn70001-bib-0008] Gobbens, R. J. , S. Vermeiren , A. Van Hoof , and T. van der Ploeg . 2022. “Nurses' Opinions on Frailty.” Healthcare (Basel, Switzerland) 10, no. 9: 1632–1646. 10.3390/healthcare10091632. 36141244 PMC9498801

[ijn70001-bib-0009] González‐Mariscal, A. , J. Corral‐Pérez , M. Á. Vázquez‐Sánchez , L. Ávila‐Cabeza‐de‐Vaca , M. Costilla , and C. Casals . 2024. “Benefits of an Educational Intervention on Functional Capacity in Community‐Dwelling Older Adults With Frailty Phenotype: A Randomized Controlled Trial.” International Journal of Nursing Studies 162: 104955–104964. 10.1016/j.ijnurstu.2024.104955. 39579605

[ijn70001-bib-0010] Gutierrez, L. , A. Folch , M. Rojas , et al. 2021. “Effects of Nutrition on Cognitive in Adults With or Without Cognitive Impairment: A Systematic Review of Randomized Controlled Clinical Trials.” Nutrients 13, no. 11: 3727–3767. 10.3390/nu13113728. 34835984 PMC8621754

[ijn70001-bib-0011] Hakeem, F. F. , E. Bernabe , and W. Sabbah . 2019. “Association Between Oral Health and Frailty: A Systematic Review of Longitudinal Studies.” Gerodontology 36, no. 3: 1–11. 10.1111/ger.12406. 31025772

[ijn70001-bib-0012] Hakeem, F. F. , E. Bernabe , and W. Sabbah . 2021. “Association Between Oral Health and Frailty Among American Older Adults.” Journal of the American Medical Directors Association 22, no. 3: 559–563. 10.1016/j.jamda.2020.07.023. 32859517

[ijn70001-bib-0013] Hao, Y. , S. Li , S. Dong , and L. Niu . 2023. “The Association Between Tooth Loss and Insulin Resistance Mediated by Diet Quality and Systemic Immunoinflammatory Index.” Nutrients 15, no. 23: 5008–5021. 10.3390/nu15235008. 38068866 PMC10708050

[ijn70001-bib-0014] Hou, T. , T. Zhang , W. Cai , et al. 2020. “Social Support and Mental Health Among Health Care Workers During Coronavirus Disease 2019 Outbreak: A Moderated Mediation Model.” PLoS ONE 15, no. 5: e0233831–e0233845. 10.1371/journal.pone.0233831. 32470007 PMC7259684

[ijn70001-bib-0015] Hsu, Y. H. , M. Y. Chou , C. S. Chu , et al. 2019. “Predictive Effect of Malnutrition on Long‐Term Clinical Outcomes Among Older Men: A Prospectively Observational Cohort Study.” Journal of Nutrition, Health & Aging 23: 876–882. 10.1007/s12603-019-1246-2.31641739

[ijn70001-bib-0016] Huang, C. H. , K. Okada , E. Matsushita , et al. 2020. “Sex‐Specific Association Between Social Frailty and Diet Quality, Diet Quantity, and Nutrition in Community‐Dwelling Elderly.” Nutrients 12, no. 9: 2845–2858. 10.3390/nu12092845. 32957506 PMC7551288

[ijn70001-bib-0017] Huang, T. Y. , M. Y. Chou , C. K. Liang , Y. T. Lin , R. Y. Chen , and P. F. Wu . 2023. “Physical Activity Plays a Crucial Role in Multidomain Intervention for Frailty Prevention.” Aging Clinical and Experimental Research 35, no. 6: 1283–1292. 10.1007/s40520-023-02412-z. 37101084 PMC10132799

[ijn70001-bib-0018] Iwasaki, M. , A. Yoshihara , M. Sato , et al. 2018. “Dentition Status and Frailty in Community‐Dwelling Older Adults: A 5‐Year Prospective Cohort Study.” Geriatrics & Gerontology International 18, no. 2: 256–262. 10.1111/ggi.13170. 28944598

[ijn70001-bib-0019] Kang, J. , B. Wu , D. Bunce , M. Ide , S. Pavitt , and J. Wu . 2019. “Cognitive Function and Oral Health Among Ageing Adults.” Community Dentistry and Oral Epidemiology 47, no. 3: 259–266. 10.1111/cdoe.12452. 30838683

[ijn70001-bib-0020] Kang, M. , and H. W. Jung . 2022. “Association Between Oral Health and Frailty in Older Korean Population: A Cross‐Sectional Study.” Clinical Interventions in Aging 17: 1863–1872. 10.2147/CIA.S384417. 36575660 PMC9790170

[ijn70001-bib-0021] Kasa, A. S. , P. Drury , V. Traynor , S. C. Lee , and H. R. Chang . 2023. “The Effectiveness of Nurse‐Led Interventions to Manage Frailty in Community‐Dwelling Older People: A Systematic Review.” Systematic Reviews 12, no. 1: 182–198. 10.1186/s13643-023-02335-w. 37777786 PMC10543273

[ijn70001-bib-0022] Kasa, A. S. , V. Traynor , and P. Drury . 2024. “Measuring the Effects of Nurse‐Led Frailty Intervention on Community‐Dwelling Older People in Ethiopia: A Quasi‐Experimental Study.” BMC Geriatrics 24, no. 1: 384–396. 10.1186/s12877-024-04909-2. 38689218 PMC11061989

[ijn70001-bib-0023] Kim, H. , E. Lee , and S. W. Lee . 2022. “Association Between Oral Health and Frailty: Results From the Korea National Health and Nutrition Examination Survey.” BMC Geriatrics 22, no. 1: 369–378. 10.1186/s12877-022-02968-x. 35477396 PMC9044774

[ijn70001-bib-0024] Komatsu, R. , K. Nagai , Y. Hasegawa , et al. 2021. “Association Between Physical Frailty Subdomains and Oral Frailty in Community‐Dwelling Older Adults.” International Journal of Environmental Research and Public Health 18, no. 6: 2931–2940. 10.3390/ijerph18062931. 33809322 PMC8001836

[ijn70001-bib-0025] Krzymińska‐Siemaszko, R. , S. Tobis , M. Lewandowicz , and K. Wieczorowska‐ Tobis . 2020. “Comparison of Four Sarcopenia Screening Questionnaires in Community‐Dwelling Older Adults From Poland Using Six Sets of International Diagnostic Criteria of Sarcopenia.” PLoS ONE 15, no. 4: e0231847–e0231863. 10.1371/journal.pone.0231847. 32310992 PMC7170245

[ijn70001-bib-0026] Kuo, Y. W. , M. Y. Chen , L. C. Chang , and J. D. Lee . 2021. “Oral Health as a Predictor of Physical Frailty Among Rural Community‐Dwelling Elderly in an Agricultural County of Taiwan: A Cross‐Sectional Study.” International Journal of Environmental Research and Public Health 18, no. 18: 9805–9815. 10.3390/ijerph18189805. 34574726 PMC8464879

[ijn70001-bib-0027] Lei, Z. , D. Qingyi , G. Feng , W. Chen , R. S. Hock , and W. Changli . 2009. “Clinical Study of Mini‐Nutritional Assessment for Older Chinese Inpatients.” Journal of Nutrition, Health & Aging 13, no. 10: 871–876. 10.1007/s12603-009-0244-1.19924346

[ijn70001-bib-0028] Li, S. , G. Cui , K. Jørgensen , Z. Cheng , Z. Li , and H. Xu . 2022. “Psychometric Properties and Measurement Invariance of the Chinese Version of the Brief Assessment of Impaired Cognition Questionnaire in Community‐Dwelling Older Adults.” Frontiers in Public Health 10: 908827–908836. 10.3389/fpubh.2022.908827. 35784243 PMC9247353

[ijn70001-bib-0029] Liu, X. , X. Xia , F. Hu , et al. 2021. “Nutrition Status Mediates the Association Between Cognitive Decline and Sarcopenia.” Aging (Albany NY) 13, no. 6: 8599–8610. 10.18632/aging.202672. 33714959 PMC8034889

[ijn70001-bib-0030] Lochlainn, M. N. , N. J. Cox , T. Wilson , et al. 2021. “Nutrition and Frailty: Opportunities for Prevention and Treatment.” Nutrients 13, no. 7: 2349–2369. 10.3390/nu13072349. 34371858 PMC8308545

[ijn70001-bib-0031] Lochlainn, M. N. , and S. Robinson . 2021. “UK Nutrition Reasearch Partnership Workshop: Nutrition and Frailty‐Opportunities for Prevention and Treatment.” Nutrition Bulletin 47, no. 1: 123–129. 10.1111/nbu.12538. 36045087

[ijn70001-bib-0032] Ma, W. B. , B. Wu , X. Q. Gao , and R. Y. Zhong . 2022. “Association Between Frailty and Cognitive Function in Older Chinese People: A Moderated Mediated of Social Relationship and Depressive Symptoms.” Journal of Affective Disorders 316: 223–232. 10.1016/j.jad.2022.08.032. 35988782

[ijn70001-bib-0033] Nagai, K. , K. Tamaki , H. Kusunoki , et al. 2020. “Physical Frailty Predicts the Development of Social Frailty: A Prospective Cohort Study.” BMC Geriatrics 20, no. 1: 403–411. 10.1186/s12877-020-01814-2. 33054731 PMC7557012

[ijn70001-bib-0034] Nagatani, M. , T. Tanaka , B. K. Son , et al. 2023. “Oral Frailty as a Risk Factor for Mild Cognitive Impairment in Community‐Dwelling Older Adults: Kashiwa Study.” Experimental Gerontology 172: 112075–112081. 10.1016/j.exger.2022.112075. 36581224

[ijn70001-bib-0035] Nangle, M. R. , J. Riches , S. A. Grainger , N. Manchery , P. S. Sachdev , and J. D. Henry . 2019. “Oral Health and Cognitive Function in Older Adults: A Systematic Review.” Gerontology 65, no. 6: 659–672. 10.1159/000496730. 30904915

[ijn70001-bib-0036] Qi, X. , Z. Zhu , B. L. Plassman , and B. Wu . 2021. “Dose‐Response Meta‐Analysis on Tooth Loss With the Risk of Cognitive Impairment and Dementia.” Journal of the American Medical Directors Association 22, no. 10: 2039–2045. 10.1016/j.jamda.2021.05.009. 34579934 PMC8479246

[ijn70001-bib-0037] Qin, Y. , J. X. Li , M. McPhillips , N. Lukkahatai , F. Yu , and K. Li . 2021. “Association of Fear of Falling With Frailty in Community‐Dwelling Older Adults: A Cross‐Sectional Study.” Nursing & Health Sciences 23, no. 2: 516–524. 10.1111/nhs.12840. 33825287 PMC8217326

[ijn70001-bib-0038] Registered Nurses' Association of Ontario . 2020. “Oral Health: Supporting Adults Who Require Assistance[EB/OL].[2024‐12‐20].” https://RNAO.ca/bpg/guidelines/oral‐health‐supporting‐adults‐who‐require‐assistance‐second‐edition.

[ijn70001-bib-0039] Rezaei‐Shahsavarloo, Z. , F. Atashzadeh‐Shoorideh , R. J. J. Gobbens , A. Ebadi , and G. G. Harouni . 2020. “The Impact of Interventions on Management of Frailty in Hospitalized Frail Older Adults: A Systematic Review and Meta‐Analysis.” BMC Geriatrics 20: 526–543. 10.1186/s12877-020-01935-8. 33272208 PMC7712609

[ijn70001-bib-0040] Saarela, R. K. T. , K. Hiltunen , H. Kautiainen , H. M. Roitto , P. Mäntylä , and K. H. Pitkälä . 2021. “Oral Health and Frailty Among Older Long‐Term Care Residents in Finland.” Journal of the American Medical Directors Association 22, no. 11: 2394–2395. 10.1016/j.jamda.2021.05.027. 34146518

[ijn70001-bib-0041] Sharifi, F. , M. A. Khoiee , R. Aminroaya , et al. 2021. “Studying the Relationship Between Cognitive Impairment and Frailty Phenotype: A Cross‐Sectional Analysis of the Bushehr Elderly Health (BEH) Program.” Journal of Diabetes and Metabolic Disorders 20, no. 2: 1229–1237. 10.1007/s40200-021-00847-7. 34900774 PMC8630203

[ijn70001-bib-0042] Shin, H. S. 2020. “The Number of Teeth Is Associated With Diet Quality in Korean Adult Population.” Archives of Oral Biology 118: 104882–104888. 10.1016/j.archoralbio.2020.104882. 32835987

[ijn70001-bib-0043] Smith, K. L. , and C. E. Greenwood . 2008. “Weight Loss and Nutritional Considerations in Alzheimer Disease.” Journal of Nutrition for the Elderly 27, no. 3–4: 381–403. 10.1080/01639360802265939. 19042581

[ijn70001-bib-0044] Song, X. Y. , W. H. Zhang , C. Hallensleben , et al. 2020. “Associations Between Obesity and Multidimensional Frailty in Older Chinese People With Hypertension.” Clinical Interventions in Aging 15: 811–820. 10.2147/CIA.S234815. 32606623 PMC7294100

[ijn70001-bib-0045] Su, Y. , M. Yuki , K. Hirayama , M. Sato , and T. F. Han . 2020. “Denture Wearing and Malnutrition Risk Among Community‐Dwelling Older Adults.” Nutrients 12, no. 1: 151–163. 10.3390/nu12010151. 31948104 PMC7020032

[ijn70001-bib-0046] Toyama, N. , D. Ekuni , D. Matsui , et al. 2021. “Comprehensive Analysis of Risk Factors for Periodontitis Focusing on the Saliva Microbiome and Polymorphism.” International Journal of Environmental Research and Public Health 18, no. 12: 6430–6442. 10.3390/ijerph18126430. 34198553 PMC8296229

[ijn70001-bib-0047] Turnbull, N. , P. Cherdsakul , S. Chanaboon , D. Hughes , and K. Tudpor . 2022. “Tooth Loss, Cognitive Impairment and Fall Risk: A Cross‐Sectional Study of Older Adults in Rural Thailand.” International Journal of Environmental Research and Public Health 19, no. 23: 16015–16024. 10.3390/ijerph192316015. 36498085 PMC9735973

[ijn70001-bib-0048] Wang, Y. , T. Xie , and J. Xu . 2022. “Family Socioeconomic Status and Internalizing Problem Behavior Among Chinese Adolescents: The Chain Mediation Effect of Academic Performance and Peer Conflict.” Frontiers in Psychology 13: 902545–902556. 10.3389/fpsyg.2022.902545. 35814078 PMC9260152

[ijn70001-bib-0049] Winkelmann, J. , R. J. Gomez , and E. van Ginneken . 2022. “Oral Health Care in Europe: Financing, Access and Provision.” Health Systems in Transition 24, no. 2: 1–176. 35833482

[ijn70001-bib-0050] Wu, J. J. , S. C. Weng , C. K. Liang , et al. 2020. “Effects of Kidney Function, Serum Albumin and Hemoglobin on Dementia Severity in the Oldest Old People With Newly Diagnosed Alzheimer's Disease in a Residential Aged Care Facility: A Cross‐Sectional Study.” BMC Geriatrics 20, no. 1: 391–401. 10.1186/s12877-020-01789-0. 33028210 PMC7541276

[ijn70001-bib-0051] Xia, X. , Z. G. Xu , F. J. Hu , L. Hou , G. C. Zhang , and X. L. Liu . 2022. “Nutrition Mediates the Relationship Between Number of Teeth and Sarcopenia: A Pathway Analysis.” BMC Geriatrics 22, no. 1: 649–660. 10.1186/s12877-022-03350-7. 35941556 PMC9360705

[ijn70001-bib-0052] Xiong, J. , and W. X. Xue . 2022. “The Role of Vitamin D in the Link Between Physical Frailty and Cognitive Function: A Mediation Analysis in Community‐Dwelling Chinese Older Adults.” Frontiers in Nutrition 9: 922673–922682. 10.3389/fnut.2022.922673. 35958260 PMC9359101

[ijn70001-bib-0053] Xu, X. , Y. Zhao , B. Wu , Y. Pei , and D. Gu . 2023. “Association Between Tooth Loss and Frailty Among Chinese Older Adults: The Mediating Role of Dietary Diversity.” BMC Geriatrics 23, no. 1: 668–678. 10.1186/s12877-023-04355-6. 37848821 PMC10583397

[ijn70001-bib-0054] Yang, H. L. , F. R. Li , P. L. Chen , X. Cheng , C. Mao , and X. B. Wu . 2022. “Tooth Loss, Denture Use, and Cognitive Impairment in Chinese Older Adults: A Community Cohort Study.” Journals of Gerontology. Series A, Biological Sciences and Medical Sciences 77, no. 1: 180–187. 10.1093/gerona/glab056. 33674815

[ijn70001-bib-0055] Yu, W. H. , W. H. Yu , X. T. Liu , et al. 2021. “Associations Between Malnutrition and Cognitive Impairment in an Elderly Chinese Population: An Analysis Based on a 7‐Year Database.” Psychogeriatrics: The Official Journal of the Japanese Psychogeriatric Society 21, no. 1: 80–88. 10.1111/psyg.12631. 33207393

[ijn70001-bib-0056] Yun, J. H. , S. K. Ki , J. Kim , D. Y. Chon , S. Y. Shin , and Y. H. Lee . 2020. “Relationships Between Cognitive Function and Frailty in Older Korean Adults: The Moderating Effect of the Number of Teeth.” Archives of Gerontology and Geriatrics 91: 104213–104220. 10.1016/j.archger.2020.104213. 32805701

[ijn70001-bib-0057] Zegeye, A. F. , Y. Z. Temachu , and C. K. Mekonnen . 2023. “Prevalence and Factors Associated With Diabetes Retinopathy Among Type 2 Diabetic Patients at Northwest Amhara Comprehensive Specialized Hospitals, Northwest Ethiopia 2021.” BMC Ophthalmology 23, no. 1: 9–17. 10.1186/s12886-022-02746-8. 36604682 PMC9814297

[ijn70001-bib-0058] Zhang, X. M. , J. Jiao , J. Cao , and X. J. Wu . 2022. “The Association Between the Number of Teeth and Frailty Among Older Nursing Home Residents: A Cross‐Sectional Study of the CLHLS Survey.” BMC Geriatrics 22, no. 1: 1007–1018. 10.1186/s12877-022-03688-y. 36585614 PMC9805096

[ijn70001-bib-0059] Zhang, X. M. , X. Wu , and W. Chen . 2022. “The Association Between Number of Teeth and Cognitive Frailty in Older Adults: A Cross‐Sectional Study.” Journal of Nutrition, Health & Aging 26, no. 5: 430–438. 10.1007/s12603-022-1783-y.35587754

[ijn70001-bib-0060] Zhao, J. , W. S. Qu , X. R. Zhou , et al. 2021. “Sleep Quality Mediates the Association Between Cerebral Small Vessel Disease Burden and Frailty: A Community‐Based Study.” Frontiers in Aging Neuroscience 13: 751369–751376. 10.3389/fnagi.2021.751369. 34744691 PMC8564177

[ijn70001-bib-0061] Zhao, X. X. , Q. Chen , Z. Liang , et al. 2022. “Longitudinal Relationship Between Frailty and Cognitive Impairment in Chinese Older Adults: A Prospective Study.” Journal of Applied Gerontology 41, no. 12: 2490–2498. 10.1177/07334648221118352. 36031943

[ijn70001-bib-0062] Zheng, L. , X. Li , Y. Qiu , et al. 2024. “Effects of Nurse‐Led Interventions on the Physical and Mental Health Among Pre‐Frail or Frail Older Adults: A Systematic Review.” Ageing Research Reviews 100: 102449–102459. 10.1016/j.arr.2024.102449. 39111408

